# Comparative Analysis of Grape Seed Oil, Linseed Oil, and a Blend: In Vivo Effects of Supplementation

**DOI:** 10.3390/foods13142283

**Published:** 2024-07-20

**Authors:** Carolina Di Pietro Fernandes, Arnildo Pott, Priscila Aiko Hiane, Valter Aragão do Nascimento, Wander Fernando de Oliveira Filiú, Lincoln Carlos Silva de Oliveira, Eliana Janet Sanjinez-Argandoña, Leandro Fontoura Cavalheiro, Carlos Eduardo Domingues Nazário, Anderson Rodrigues Lima Caires, Flavio Santana Michels, Karine de Cássia Freitas, Marcel Arakaki Asato, Juliana Rodrigues Donadon, Danielle Bogo, Rita de Cássia Avellaneda Guimarães

**Affiliations:** 1 Graduate Program in Health and Development in the Central-West Region of Brazil, Experimental Disease Models Laboratory (LMED-Finep), Medical School, Federal University of Mato Grosso do Sul, Campo Grande 79070-900, Brazil; nutricaroldipietro@gmail.com (C.D.P.F.); priscila.hiane@ufms.br (P.A.H.); aragao60@hotmail.com (V.A.d.N.); karine.freitas@ufms.br (K.d.C.F.); danielle.bogo@ufms.br (D.B.); 2 Laboratory of Botany, Institute of Biosciences, Federal University of Mato Grosso do Sul, Campo Grande 79070-900, Brazil; arnildo.pott@gmail.com; 3 Pharmaceutical Science, Food and Nutrition Faculty, Federal University of Mato Grosso do Sul, Campo Grande 79070-900, Brazil; wander.filiu@gmail.com (W.F.d.O.F.); juliana.donadon@ufms.br (J.R.D.); 4 Chemistry Institute, Federal University of Mato Grosso do Sul, Campo Grande 79070-900, Brazil; lincoln.oliveira@ufms.br (L.C.S.d.O.); lernfc@gmail.com (L.F.C.); carlos.nazario@ufms.br (C.E.D.N.); 5 School of Engineering (FAEN), Federal University of Grande Dourados (UFGD), Cidade Universitária, Dourados-Itahum Road 7 Km 12, Dourados 79804-970, Brazil; elianaargadona@ufgd.edu.br; 6 Optics and Photonics Group, Institute of Physics, Federal University of Mato Grosso do Sul, Campo Grande 79070-900, Brazil; anderson.caires@ufms.br (A.R.L.C.); flavio.michels@ufms.br (F.S.M.); 7 Medical School, Federal University of Mato Grosso do Sul, Campo Grande 79070-900, Brazil; marcel_arakakiasato@hotmail.com

**Keywords:** fatty acids, linolenic acid, seed oil, alpha-linolenic acid, cytokines

## Abstract

Grape seeds are rich in bioactive substances, including polyphenols, terpenoids, and phytosterols. Linseed (*Linum usitatissimum* L.) boasts a high concentration of polyunsaturated fatty acids (PUFAs), lignans, phytoestrogens, and soluble fibers, all contributing to its therapeutic potential. In this study, we pioneered the formulation of an oil blend (GL) combining grape seed oil (G) and golden linseed oil (GL) in equal volumes (1:1 (*v*/*v*)) and we evaluated in terms of the nutritional, physical, and chemical properties and their influence in an in vivo experimental model. We analyzed the oils by performing physical–chemical analyses, examining the oxidative stability using Rancimat; conducting thermal analyses via thermogravimetry/derivative thermogravimetry (TG/DTG) and differential scanning calorimetry (DSC), performing optical UV–vis absorption analyses; examining the fluorescence emission–excitation matrix, total carotenoids, and color, and conducting metabolic assessments in an in vivo experimental trial. The fatty acid profile presented a higher fraction of linoleic acid (C18:2) in G and GL and alpha-linolenic acid (C18:3) in L. The acidity and peroxide indices were within the recommended ranges. The TG/DTG, DSC, and Rancimat analyses revealed similar behaviors, and the optical analyses revealed color variations caused by carotenoid contents in L and GL. In the in vivo trial, G (G2: 2000 mg/kg/day) promoted lower total consumption, and the blend (GL: 2000 mg/kg/day) group exhibited less weight gain per gram of consumed food. The group with G supplementation (G2: 2000 mg/kg/day) and GL had the highest levels of HDL-c. The group with L supplementation (L2: 2000 mg/kg/day) had the lowest total cholesterol level. The L2, G1 (1000 mg/kg/day), and G2 groups exhibited the lowest MCP-1 and TNF-α values. Additionally, the lowest adipocyte areas occurred in G and GL. Our results suggest that this combination is of high quality for consumption and can influence lipid profiles, markers of inflammation, and antioxidant status.

## 1. Introduction

Vineyards of the grape species *Vitis vinifera* L. have a high economic impact, and they are traditionally grown in temperate regions, primarily those in Europe, northern Africa, and West Asia [1].

Grape seeds are rich in bioactive substances, including polyphenols, terpenoids, and phytosterols, and other compounds [2]. Due to their chemical profile, they have potential applications in the pharmaceutical and food industries, ranging from oil extraction to the developing food supplements and cosmetic products [3].

The seeds are byproducts of the vine and juice industry, and they are generally discarded during the production processes [3]. There is a growing interest in grape residues and techniques, aiming to achieve the full use of foods because studies have emphasized the therapeutic properties of the stems, peels, seeds, and leaves [4]. Extracted grape seed oil (*V. vinifera*) represents 6–20% of the seed composition [5], and extraction via cold pressing ensures the highest quality as well as preservation of its antioxidant properties [4,6].

The grape seed oil composition presents an average of 90% unsaturated fatty acids, with linoleic acid (65–75%) and oleic acid (20–40%) being among them, and only 10% saturated fatty acids [5]. Additionally, it demonstrates inflammatory action on reactive species of oxygen (RSO) and on the reduction in inflammatory markers, such as the secretion of TNF-α, IL-1β, and IL-6, as well as other pro-inflammatory molecules, acting in the health–disease process [5].

Linseed (*Linum usitatissimum* L.), another well-researched seed, is renowned for the quality of its oil, which boasts a high concentration of polyunsaturated fatty acids (PUFAs), accounting for 73% of its composition. Notably, it contains 60% alpha-linolenic acid (C18:3), besides a significant level of monounsaturated fatty acids (MUFAs) at 18%. Additionally, linseed is a rich source of lignans, phytostroegens, and soluble fibers, all of which contributing to its therapeutic potential [7,8]. Linseed oil is also obtained via cold pressing and contains only 9% saturated fatty acids, like grape seed oil [5,6].

Linseed oil’s composition is instrumental in regulating blood lipid levels, helping to lower total cholesterol, low-density lipoprotein (LDL-c), and serum triglycerides [8]. It also plays a role in reducing tissue inflammation caused by oxidative stress and decreasing the occurrence of primary cardiovascular events due to its high concentration of α-linolenic acid [9].

There is a consensus that a healthy diet with a moderate consumption of vegetable oils preferred over animal fats because oils extracted from seeds improve metabolic profiles, such as glycemia and serum lipoproteins, and reduce inflammatory cytokines. The diet also has various components such as a fatty acid profile with a high concentration of MUFAs and PUFAs, relevant to human health [10].

Nutritional interventions are the best option compared with the high cost and adverse effects of drugs [11,12], with the incorporation of protective foods acting on the metabolism being crucial. Therefore, an evaluation of the bioavailability of the nutraceutical substances present in seed oils is necessary, as different degrees of fruit ripening, in addition to soil fertility, climate, and temperature, among other influences of natural origin, impact the nutritional quality of the seeds and their extracted oils [13].

Considering these facts, linseed oil and grape seed oil, each possessing distinct fatty acid profiles and unique nutritional, physical, and chemical properties, present an intriguing opportunity for blending [14,15]. Combining these oils in equal proportions (1:1, *v*/*v*) creates a composition that does not occur naturally. Therefore, conducting a comprehensive assessment of grape seed oil, linseed oil, and their blended mixture in terms of nutritional and physicochemical properties is crucial [14,15]. Additionally, extended investigations into their compounds and impacts on in vivo experimental models are essential. Furthermore, predicting conditions for their application can contribute to economic growth and advancements in nutrition and technology [16].

## 2. Materials and Methods

### 2.1. Raw Material 

In this study, cold-pressed vegetable oils from seeds of *Vitis vinifera* L. *and Linum usitatissimum* L. seeds were sourced from reputable suppliers at Indústria Pazze Alimentos™, Panambi (RS), Brazil. These oils were obtained by using the cold pressing technique, ensuring low acidity and peroxide indices, as verified by the manufacturer. This process preserved the oils’ purity and uniformity, which are critical for the integrity of this research.

To develop a consistent oil blend, the meticulous blending of equal volumes of grape seed oil and golden linseed oil was conducted using volumetric flasks to establish a precise 1:1 (*v*/*v*) ratio. Delicate agitation was employed to avoid introducing of air bubbles that could compromise the blend’s stability. Subsequently, the oil blend was stored in an airtight and sterile container, shielded from direct solar exposure, and kept at a stable temperature to preserve its quality and inhibit oxidative deterioration.

Extra virgin olive oil of the Andorinha Portugal™ Ferreira do Alentejo, Portugal brand was used in this study for the oil-supplemented control group, with its identity and quality details specified on the label.

### 2.2. Fatty Acid Profile

Fatty acids were esterified using a method adapted from Maya and Rodriguez-Amaya (1993). The resulting fatty acid methyl esters (FAMEs) were analyzed via gas chromatography (GC 2010, Shimadzu, Tokyo, Japan) to identify their peaks. The FAME standard consisted of a mixture of 37 components that mimic the fatty acid composition found in many food samples. FAMEs characterize the lipid fraction in foods and determine the total fat and trans fat contents. This technique involves the separation of fatty acids on a gas chromatography column [17].

The apparatus featured a flame ionization detector (FID) and a BPX-70 capillary column with precise dimensions and film thickness. Both the injector and detector were heated to 250 °C. Initially, the column temperature was held at 80 °C for three minutes, increased to 140 °C at 10 °C/min, and finally raised to 240 °C at 5 °C/min, where it remained for five minutes. By comparing the relative retention times to those of a Supelco C22 FAME pattern (99% pure), we pinpointed the FAME peaks. The fatty acid content was quantified by integrating the areas of these peaks, and the results are presented as grams of fatty acids per gram of oil extracted.

Additionally, we utilized the atherogenicity index (Equation (1)) and the thrombogenicity index (Equation (2)) [18].

(1) $$\mathrm{Atherogenic}\;\mathrm{index}=\frac{\;\left[\left(C12:0+\left(4\times \;C14:0\right)+C16:0\right)\right]}{\left(\sum \mathrm{MUFA}\;+\sum \omega 6+\sum \omega 3\right)}$$

(2) $$\mathrm{Thrombogenicity}\;\mathrm{index}=\frac{\left(C14:0+\;C16:0+\;C18:0\right)}{\left[\left(0.5\times \sum \mathrm{MUFA}\;\right)+\left(0.5\times \sum \omega 6\right)+\left(3\times \sum \omega 3\right)+\left(\omega 3/\omega 6\right)\right]\;}$$

Here, MUFA is the sum of the monounsaturated fatty acids of the studied oils. 

### 2.3. Basic Quality Parameters and Chemical–Physical Analyses

#### 2.3.1. Acidity Index 

The oil acidity index was determined by adding a neutralized ether–alcohol (1:1) solution and phenolphthalein to indicate color change. We utilized potassium hydroxide (KOH) 0.1 N as a titrant until the color changed to pink. The results are expressed as mgKOH/g and g of oleic acid/100 g oil [19].

#### 2.3.2. Peroxide Index

The peroxide value was assessed by adding 5.0 mL of an acetic acid–chloroform solution (3:2), 0.1 mL of a saturated potassium iodide solution, and 0.1 mL of a 1% soluble starch solution as an indicator to the oils. The mixture was then allowed to stand, shielded from light. Titration was conducted using a 0.01 N sodium thiosulfate solution, and the results are expressed as milliequivalents of oxygen per kilogram (mEqO_2_/kg) [18,20]. 

#### 2.3.3. Refraction Index

The refraction index of the oils was obtained with an Abbé refractometer (RL3, Tecnal, Ourinhos, Brazil) calibrated with distilled water, with a refraction index of 13.330, at 27 °C, temperature-corrected to 40 °C [18,20].

#### 2.3.4. Iodine Index

The iodine index was obtained by adding carbon tetrachloride and Wijs solution to the oils, and standard sodium thiosulphate was used as a titrant until the color changed from black to pink. The results are expressed as g I_2_/100 g [18,20].

#### 2.3.5. Saponification Index 

We determined the saponification index by adding a solution of alcohol and potassium hydroxide (KOH) at 4% (*m*/*v*) to the samples and refluxing for 1 h. Next, we used phenolphthalein to indicate color change and titration with chloridric acid (HCl) 0.5 N. The results are expressed as mgKOH/g [18,20].

#### 2.3.6. Relative Density at 25 °C/25 °C

We determined the relative density by using the pycnometer method, with prior taring in an oven at 105 °C. Then, we added a mixture of alcohol and water at 20–23 °C and placed it in a bath at a constant temperature (25 ± 0.1 °C). After 30 min, we adjusted it to the water level and weighed it on an analytic scale. We repeated the same procedures with the oils. The results are expressed as mg/mL [18,20].

### 2.4. Oxidative Stability: Rancimat

Oxidative stability was obtained through the induction period (PI) resulting from the Rancimat test, following the EN 14112 method, utilizing Rancimat equipment (893 Professional Biodiesel Rancimat, Metrohm, São Paulo, Brazil). Analyses were performed with 3.0 g of oil without dilution at 110 °C, with a constant airflow of 10 L/h through the samples, followed by a recipient containing 50 mL of deionized water, and the conductivity produced by the volatile products during the degradation of the vegetable oils was measured as a function of time [18,21].

### 2.5. Thermal Analysis

#### 2.5.1. Thermogravimetry and Derivative Thermogravimetry (TG/DTG)

For TGA/DTG curves, we utilized 4.0 mg of each oil in a TGA Q50 system (TA Instruments, Eden Prairie, MN, USA, EUA) under a nitrogen atmosphere, with a flow of 60 mL/min in the oven and a heating rate of 10 °C/min at temperatures varying between ambient and 700 °C, with platinum crucibles used as supports [21].

#### 2.5.2. Differential Scanning Calorimetry (DSC)

We evaluated the crystallization and fusion processes of the oils by generating DCS curves with a sample mass of around 3 mg in a DSC (Q20 TA Instruments, EUA) equipped with a double-stage cooling system (RCS 90). Cooling and heating curves were programmed in cycles. Initially, the temperature was equilibrated at 60 °C, followed by an isotherm of 10 min and a cooling ramp until reaching −60 °C, at a pace of 5 °C/min, completing Cycle 1. In sequence, the temperature was equilibrated at −60 °C, followed by an isotherm for 10 min and a heating ramp at 5 °C/min until the final temperature of 60 °C was reached, completing Cycle 2. The total analysis time was close to 70 min.

### 2.6. Optic Analyses

#### UV–Vis Absorption and Excitation–Emission Matrix (EEM) Fluorescence Spectroscopy

The samples were diluted in hexane (spectroscopic grade, Sigma-Aldrich, St. Louis, MO, USA, >99%) at concentrations of 1.0, 5.0, and 90 g/dm^3^. We obtained the ultraviolet and visible (UV–vis) absorption spectra in the 200 to 600 nm range with the aid of a spectrometer (UV–vis Lambda 265, PerkinElmer^®^, Shelton, CT, USA), using a quartz cuvette with an optic length of 10 mm and a capacity of 3.5 mL. We determined the excitation–emission matrix (EEM) fluorescence spectra of the samples at a concentration of 90 g/dm^3^ using a bench spectrofluorimeter (FluoroMate FS-2, Scinco^®^, Seoul, Republic of Korea). The spectrofluorimeter consisted of excitation and emission monochromators, a 150 W Xenon excitation lamp, and an R-928 PMT detector. The analyses were performed using a 90° angle geometry between excitation and emission, using a quartz cuvette with four polished faces and a 1 cm optical path length, with the excitation and emission slits set to 5 nm. All analyses were performed at room temperature.

### 2.7. Color

Colors were determined by using a portable spectrometer (Konica Minolta^®^ model CM-2300d, Tokyo, Japan). The results are expressed as L*, a*, and b*, according to the color space L* a* b* and applying the scale CIE L* a* b*, where L* represents the light, varying between 0 (without light or black) and 100 (white); a* represents the colors from red (+a*) to green (−a*); and b* represents the color range from yellow (+b*) to blue (−b*). From the results, we obtained the *Hue* angle indices (Equation (3)), and it was then possible to define the tone in degrees and chroma (C*) (Equation (4)), which indicate the color saturation of samples.

(3) Hue=b*a*

Here, the Hue value is the color tone defined in degrees, b* indicates the chromaticity on the axis varying from yellow to blue, and a* shows the chromaticity on the axis varying from red to green (Minolta Corporation™, Tokyo, Japan, 1994).

(4) C* =a*2+b*2

Here, the C* value (chroma) indicates the color saturation, b* indicates the chromaticity on the axis varying from yellow to blue, and a* shows the chromaticity on the axis varying from red to green (Minolta Corporation™, 1994).

### 2.8. Determination of Carotenoids

The samples of G, L, and GL were initially vacuum-filtered in an environment protected from light to remove possible impurities. The method was performed according to recommendations by Rodriguez-Amaya (1999) [22] and Maldonade et al. (2021), with some modifications depending on the oil purity. Several tests were conducted to define the proportions of oil and petroleum ether P.A. (dynamic) in order to obtain absorbances between 0.2 and 0.8. Thus, aliquots of 3 to 5 g of oil were mixed with 3 to 5 mL of petroleum ether P.A., and the volume was adjusted with the same solvent in a volumetric flask (10 mL) for subsequent reading on a spectrophotometer. The subsequent steps followed the methodology described by Rodriguez-Amaya (1999) [22]. We carried out all steps with aluminium foil protecting from light, avoiding carotenoid photodegradation. We performed three dilutions of each sample and conducted absorbance readings on a UV–visible spectrophotometer (Biochrom Libra S60PC) at a wavelength of 450 nm, using quartz cuvettes. We used P.A. petroleum ether as the blank. The carotenoid content was calculated using Equation (5), with a β-carotene absorptivity value of 2592. Absorbance readings were taken for each sample in triplicate, with 3 repetitions in each dilution. The result is expressed as micrograms of carotenoids per hundred grams of oil (µg/100 g).

(5) Carotenoids μgg=A×V×104E1cm1%×m

Here, *A* is the absorbance at the maximum absorption peak, *V* is the final sample volume (mL), *m* is the sample mass (g), and $E_{1\mathrm{cm}}^{1\%}$ is the extinction coefficient (β-carotene = 2592 in petroleum ether).

### 2.9. In Vivo Experiment

#### 2.9.1. Animals

We developed this study according to the ethics precepts of Law n. 11.794 of 8 October 2008, of Decree n. 6.899 of 15 July 2009, and the rules edited by the National Council of Control of Animal Experimentation (Concea) and approved by the Ethics Commission in the Use of Animals (CEUA) of UFMS (n. 1339/2022).

One hundred and twenty-six Swiss Webster strain mice (*Mus musculus*), adult males aged 12 weeks, with an average weight of 36 g, were used. They were supplied by the Central Vivarium of the Federal University of Mato Grosso do Sul. The acclimatization period occurred 7 days before the start of the experimental period. During this time, the animals were kept in an environment with a controlled temperature (71.6 ± 33.8 °F), under a 12 h light/dark cycle, with ad libitum access to food and water, with four to five animals per cage.

#### 2.9.2. Experimental Design

**Figure 1:**
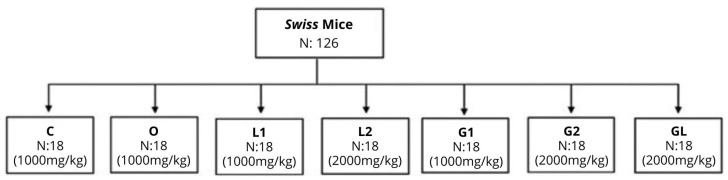
Flowchart of the experimental groups after 7 days of adaptation. C: control group (distilled water at dose of 1000 mg/kg/animal); O: extra virgin olive oil group (1000 mg/kg/animal); L1: Golden linseed oil group (1000 mg/kg/animal); L2: Golden linseed oil group (2000 mg/kg/animal); G1: Grape seed oil group (1000 mg/kg/animal); G2: Grape seed oil group (2000 mg/kg/animal); GL: blend oil (2000 mg/kg/animal).

This study included two standard control groups: one supplemented with distilled water (C) and the other supplemented with olive oil (O). Additionally, there were five experimental groups (L1, L2, G1, G2, GL). The experimental design, as depicted in Figure 1, drew inspiration from previous studies by Torres et al. (2020) [23], Marcelino et al. (2022) [24], and Silva et al. (2023) [25]. These studies utilized the gavage administration of *Caryocar brasiliense* Cambess oil, *Mauritia flexuosa* oil, and *Acrocomia aculeata* oil in *Swiss* mice at corresponding dosages of 1000 mg/kg/day and 2000 mg/kg/day, demonstrating efficacy in their results. Following the acclimation period, daily dosages were administered for 11 weeks (see Table 1 and Figure 1).

#### 2.9.3. Food Ingestion and Weight Gain

All animals received a standard commercial normocaloric diet (Nuvital^®^) ad libitum, and food consumption was measured according to the weekly consumption of the individual animals, making it possible to assess the average weekly consumption of the mice.

Supplementation was administered daily via gavage throughout the 11-week study period. Dosage adjustments for each group were performed by changes in body weight, which were assessed thrice weekly (Table 1 and Figure 1).

We evaluated food consumption and weight gain weekly, using the food efficiency coefficient (FEC) (Equation (6)) to determine the extent to which 1 g of food ingested promotes body weight gain [26].

(6) $$\mathrm{FEC}=\left(\mathrm{FW}\;-\;\mathrm{IW}\right)÷\mathrm{TFA}$$

Here, FW is the final body weight in grams, IW is the initial body weight in grams, and TFA represents the total amount of ingested food in grams [26].

We calculated the coefficient of weight gain by caloric intake (CWGCI) to measure animal weight gain in order to obtain the animal capacity to convert consumed food energy into body weight (Equation (7)).

(7) $$\mathrm{CWGCI}=\left(\mathrm{FW}\;-\;\mathrm{IW}\right)÷\mathrm{kcal}\;\mathrm{ingested}$$

Here, FW represents the final body weight in grams, IW is the initial body weight in grams, and kcal ingested is the caloric value of the ingested diet [26].

The normocaloric commercial feed (Nuvital^®^) composition is shown in Table 2, describing the ingredients, percentage of macronutrients (carbohydrates, proteins, and lipids), and number of calories per gram. 

#### 2.9.4. Euthanasia

Euthanasia was performed on the animals after a fasting period of six hours, with water available ad libitum. Anesthesia was conducted with a lethal dose of isoflurane, followed by exsanguination through the posterior vena cava for tests of serum parameters. We removed five visceral sites of fat (epidydimal, mesenteric, omental, retroperitoneal, and perirenal) and the liver, all weighed on an electronic scale (Bel Diagnóstica^®^) in milligrams (mg). The epidydimal tissue and liver were stored in 10% formaldehyde for histological analyses.

The adiposity index (%) was calculated using the formula adapted from Taylor and Phillips (1996) [27] (Equation (8)):

(8) $$\mathrm{Adiposity}\;\mathrm{Index}\;\left(\%\right)=\frac{\left(\mathrm{Sum}\;\mathrm{of}\;\mathrm{visceral}\;\mathrm{fat}\;\mathrm{sites}\right)\times 100}{\mathrm{Final}\;\mathrm{body}\;\mathrm{weight}}$$

#### 2.9.5. Serum Parameters

In this study, blood samples were collected from the posterior vena cava and transferred to tubes containing a separator gel. After centrifugation at 5000 rpm for 10 min using a refrigerated centrifuge (model universal 320, Hettich), serum was obtained. The enzymatic colorimetric methodology, with visible light reading through a green filter, was employed to determine serum parameters. Glucose (code 277), triglyceride (code 643), total cholesterol (code 167), high-density lipoprotein (HDL-c) (code 166), low-density lipoprotein (LDL-c), and very-low-density lipoprotein (VLDL-c) levels were analyzed following the manufacturer’s instructions (Labtest^®^, Lagoa Santa, Minas Gerais, Brazil).

We determined total cholesterol (TC) using the cholesterol esterase methodology. For high-density lipoprotein (HDL-c), we used the selective precipitation method with magnesium chloride to separate the other lipoproteins (VLDL-c, LDL-c, and IDL-c) and then used the cholesterol esterase method for determination. For triglycerides (TGs), we used the methodology according to Trinder—the action of lipase to isolate glycerol and the action of glycerol kinase, with hydrogen peroxide as the main product, which reacts by coupling with 4-chlorophenol and 4-amino antipyrine, with the product being anti-pyrylquinonemine, which has a red color (cherry). Fasting glycemia was measured utilizing the enzymatic colorimetric method, and for spectrophotometry measurements, the glucose oxidase method was used [28]. All analyses were performed simultaneously with quality control. 

We calculated low-density lipoprotein (VLDL-c) (Equation (9)), low-density lipoprotein (LDL-c) (Equation (10)), and cholesterol non-HDL (non-HDL-c) (Equation (11)) [28] as follows:

(9) VLDL Cholesterol Values = Triglycerides/5

(10) $$\mathrm{LDL}\;\mathrm{Cholesterol}=\;\mathrm{Total}\;\mathrm{cholesterol}\;-\left(\mathrm{HDL}-c+\mathrm{VLDL}-c\right)$$

(11) Non−HDL cholesterol= Total cholesterol − HDL−c

#### 2.9.6. Cytokines

The adipokine bead panel, which includes IL-6, MCP-1, TNF-α, PAI-1, insulin, leptin, and resistin, was quantified using the commercial kit MAD-KMAG-71K (Merck-Sigma Aldrich, São Paulo, Brazil). The plates were analyzed on a Luminex MAGPIX System (Luminex Corporation, Austin, TX, USA) and the data were generated with xPONENT software 4.3. Luminex^®^. We obtained the concentration values in pg/mL from blood serum centrifuged in a tube with a separator gel. We vortexed the serum for 30 s and centrifuged it at 6000 rpm for 10 min. Next, 10 µL of the serum of each animal was placed on a plate with 96 wells, together with 10 µL of an assay buffer solution and 25 µL of a solution containing seven adipokines. We also prepared the blank, standard, and control parameters by following the instructions (Milliplex^®^ MAP kit, Billerica, MA, USA). Afterwards, the plate was read on a Luminex^®^ using MAGPIX^®^ software, and concentration values were obtained in pg/mL.

#### 2.9.7. Histology of the Epidydimal Adipose Tissue and Liver

After euthanasia, the liver was removed for histological study and fixed in a 10% formaldehyde solution until embedded in paraffin. Then, 7 µm thick sections were introduced using a microtome, with subsequent mounting on glass slides.

To examine the treatment effects on the hepatocytes, pathologists analyzed the liver, utilizing the Kleiner et al. system (2005) [29] which evaluates the degree of steatosis (<5%, 5 to 33%, 34 to 66%, >66%), microvesicular steatosis (absent or present), lobular inflammation (absent, <1 focus/field, 2–4 foci/field, or >4 foci/field), ballooning (absent, few cells, or many cells), Mallory’s hyaline (absent or present), glycogenated nucleus (none/rare or some), and apoptosis (absent or present). The epidydimal adipose tissue area was analyzed according to the method described by Jernås et al. (2006) [30].

### 2.10. Statistical Analyses

We performed analyses utilizing the Jandel Sigma Stat software, version 3.5 (Systat Software, Inc., San Jose, CA, USA), and Sigma Plot, version 12.5 (Systat Software Inc., San Jose, CA, USA), obtaining mean ± standard deviation (DP) values. We compared the groups by utilizing an ANOVA on ranks followed by Tukey’s post-test and the Kruskal–Wallis test followed by Dunn’s test (*p* < 0.05). Histological data are described in absolute (n) and relative frequencies (%). The chi-square test was utilized to evaluate the association in the histological analysis, followed by Bonferroni correction, using the statistical program Biostat 5.0. Significance was considered at *p* < 0.05.

## 3. Results and Discussion

Individually, both grape seed oil and linseed oil show potential for enhancing human health due to their compositions, making possible the improvements in the domains of cardiovascular health and anti-inflammatory effects. Despite the anticipated scientific support for the individual use and commercialization of grape seed oil and linseed oil, the fusion of these oils (*blend*) results in an oil rich in polyunsaturated fatty acids, with thepossible presence of antioxidants and bioactive compounds derived from both oils. This blend combines the complementary profiles of both seeds. Our quality analyses, including optical techniques, lipid stability, and oxidative stability, evaluated the nutritional quality and behavior of this blend compared with those of the individual oils.

The fatty acid profile demonstrated the stability and nutritional quality of the studied oils [31]. In the analysis detailed in Table 3, we identified 14 fatty acids in the grape seed oil (G), 15 fatty acids in the linseed oil (L), and 14 fatty acids in the blend oil (GL). Polyunsaturated fatty acids (PUFAs) were the predominant type in these oils, with linoleic acid (C18:2) being the most abundant in G and GL at 52.40% and 42.53%, respectively, and alpha-linolenic acid (C18:3) constituting 53.30% of L. As for monounsaturated fatty acids (MUFAs), oleic acid (C18:1) was found to be the highest in all oils, at 27.94% in G, 25.57% in GL, and 18.31% in L. Among the saturated fatty acids, palmitic acid (C16:0) was the most prevalent, observed at 13% in G, 12.45% in GL, and 9.9% in L.

The content of total saturated fatty acids was low in all oils, with values of 13%, 9.9%, and 12.45% for G, L, and GL. The total content of unsaturated fatty acids was the highest in G, L, and GL at 82.61%, 89.63%, and 86.86%, respectively. The atherogenicity and thrombogenicity indices were similar between the oils. The content of total saturated fatty acids was low in all oils, with values of 13%, 9.9%, and 12.45% for G, L, and GL. The total content of unsaturated fatty acids was the highest in G, L, and GL at 82.61%, 89.63%, and 86.86%, respectively. The atherogenicity and thrombogenicity indices were similar between the oils. 

Grape seed oil (G) is rich in polyunsaturated fatty acids, mainly linoleic acid (C18:2) (Table 3). In our study, G was composed of a mix of *Vitis vinifera* seeds, resulting in a percentage of linoleic fatty acid (C18:2) between those of the varieties Sangiovese (47.34%) and Cinsaut (72.91%) [14,31]. The tested grape seed oil (Table 3) had a higher presence of oleic fatty acid (C18:1) than 10 grape varieties (Chardonnay, Muscadine, Rubi, Sangiovese, Concord, Ada-karası, Cinsaut, Cabernet Sauvignon, Gamay, and Narince), with the oleic acid (C18:1) content ranging between 13.35% (Concord) and 26.30% (Sangiovese) [14,31]. The total unsaturated fatty acid content in the grape seed oil (82.61%) (Table 3) is comparable to the result of 82.35% reported by Hussein and Abdrabba (2015) [32].

Other vegetable oils are sources of mono- and PUFAs, such as L, which had a total unsaturated fatty acid content of 89.63% (Table 3), with the polyunsaturated alpha-linolenic acid constituting 53.3% of this fraction; this level is within the reported variation of 45% to 55% [33]. Thus, the oleic acid (C18:1) content in L (Table 3) agrees with the range of 12% to 30% (C18:1) found in other varieties [7,33]. Additionally, L had a total saturated fatty acid content of 9.9% (Table 3), within the expected value of <11% [7], having the lowest percentage of total saturated fatty acids among the studied oils.

The blend oil (GL) exhibited a fatty acid profile that was intermediate compared to those of the grape seed (*V. vinifera*) and linseed (*Linum usitatissimum*) oils. GL had an unsaturated fatty acid content of 86.86%, with notable amounts of the polyunsaturated fatty acids linoleic acid (C18:2) and alpha-linolenic acid (C18:3), alongside the presence of oleic acid (C18:1) and a total saturated fatty acid content of 12.45% (Table 3). G had a higher concentration of oleic acid (C18:1) (52.40%) than GL and L, but its concentration was lower than that of peanut oil and almond oil [14]. Around 10% of the total contents of saturated fatty acids in G, L, and GL are in congruence with those in other reports, with high levels of palmitic acid (C16:0), followed by stearic acid (C18:0), in all tested oils [34].

Linseed oil and grape seed oil, each with distinct fatty acid profiles, offer a compelling opportunity for blending. Linseed oil boasts a high content of alpha-linolenic acid (ALA), an essential omega-3 fatty acid. In contrast, grape seed oil is rich in linoleic acid, an omega-6 fatty acid. Blending these oils in equal proportions creates a novel composition that nature does not provide and that has a unique fatty acid profile, potential health advantages, culinary versatility, and use in supplementation [10,12]

MUFAs and PUFAs acquired from food affect cell and tissue responsivity, regulating antioxidant signaling pathways and modulating inflammatory processes [35]. These fatty acids are energy sources; they compose the cell membrane phospholipids and present signaling specific to hormone interactions [36]. Therefore, the moderate consumption of vegetable oils rich in MUFAs and PUFAs is recommended to prevent cardiovascular and metabolic diseases, such as type 2 diabetes, inflammatory conditions, and cancer [35,36].

Two leading families of PUFAs are relevant to health: omega-3 and omega-6. In most diets, these fatty acids are obtained from plants, such as seeds, nuts, and vegetable oils [36]. α-linolenic (C18:3) and linoleic fatty acids (C18:2) participate in intracellular signaling, such as in transcription factors and gene expression [35]. Therefore, a deficiency of essential fatty acids causes dermatitis, disturbances in mitochondrial activity, cardiovascular diseases, cognitive deficits, arthritis, and other health conditions [37].

The metabolic conversion of linoleic fatty acid (C18:2) into arachidonic acid (C20:4) and α-linolenic acid (C18:3) in eicosapentaenoic acids (C20:5) occurs through the same enzymes; therefore, a high presence of these fatty acids in an organism relates to substrate availability [38]. Linoleic acid (C18:2) acts as a substrate for the synthesis of γ-linolenic acid (C18:3) and dihomo-γ-linolenic acid (C20:3), the same way that it incorporates phospholipids into the cell membrane [38].

Table 3 describes the atherogenicity index (AI) and the thrombogenicity index (TI) for the oils studied. There are no recommended values for these indices; however, their low values across all oils indicate the quality of the fatty acids, with proportions favorable to vascular health. This demonstrates the absence of correlations with atherosclerotic and thrombogenic events [33].

The quality and identity indices of the oils are shown in Table 4. The acidity index was the highest in L (2.8 mg KOH/g), followed by GL (1.3 mg KOH/g) and G (1.2 mg KOH/g). G presented the highest level of peroxides (6.5 mEqO_2_/kg), followed by GL (4.6 mEqO_2_/kg), while L had the lowest peroxide content (2.0 mEqO_2_/kg). The refraction index values were 1.470 and 1.477 in G and L, respectively. GL exhibited results between the G and L oils because of its composition being 1:1 (*v*/*v*) (Table 4). The iodide index and relative density of G (122.71 and 0.910, respectively) were closer to those of GL (128.59 and 0.918) than those of L (175 and 0.927, respectively). GL was similar to G in terms of the saponification index and was close to L.

The quality and identity of G, L, and GL (Table 4) fall within the recommended acidity index for cold-pressed oils (<4.0 mg KOH/g), indicating low levels of free fatty acids and the absence of deterioration [39]. The peroxide index is related to the presence or absence of hydroperoxides, which form in the initial steps of lipid auto-oxidation [39]. For this index (Table 4), all samples are within the limit recommended for cold-pressed virgin oils (<15 mEqO_2_/kg), indicating the absence of oxidative processes in the analyzed oils.

These results can be attributed to the antioxidant components in G, L, and GL, such as the phenolic compounds, carotenoids, and tocopherols, which help prevent oxidative events, thus improving oil stability [40].

The refraction index is related to the viscosity of the vegetable oil, and high indices indicate a deteriorated product or a deterioration process [39]. Concerning the refraction index, the grape seed oil and linseed oil are within the recommended ranges of 1.467–1.477 and 1.472–1.487, respectively [40]. The blend oil has an index between the grape seed oil and the linseed oil (Table 4) because of its composition being 1:1 (*v*/*v*), showing coherence with the integrity of its component oils and values within the recommended range for grape seed oil and linseed oil.

The iodine index reveals the unsaturations of fatty acids in vegetable oils and their tendency to oxidize; thus, the higher the number of unsaturations, the higher the predisposition to oxidation [24,39]. G presented an iodine index below the expected range of 128–150 g I_2_/100 g [40]. Variations in this index are related to the fatty acid profile of different varieties of grapes (*V. vinifera*), which result in the extracted oils having individual characteristics [9,14]. Therefore, the literature offers figures above the recommendations, e.g., 165.5 I_2_/100 g and 194 I_2_/100 g [32,37], which, observed together with other analyses, result in an oil with good stability [41]. L and GL have iodine indices within the indicated range of 170–211 I_2_/100 g [7], similar to the cold-pressed G [3,24]. 

The saponification index indicates the presence of low-molecular-weight fatty acids in oils, inversely proportional to the presence of high-molecular-weight (long-chain) fatty acids. The obtained saponification indices (Table 4) were within the limits of 188–194 mg KOH/g for G and 185–197 mg KOH/g for L [40], and GL stayed within the recommended range. Thus, the levels of low-molecular-weight fatty acids were within the indicated patterns for all samples.

In contrast, the relative density of G (Table 4) was below the recommended range of 0.920 to 0.926 mg/mL, while that of L fell within the expected range of 0.925 to 0.935 mg/mL. However, GL had a value of 0.918 mg/mL, which is below the recommended limits for both G and L [40].

Some analyses showed results divergent from those in the literature because the specific cultivars of the tested oils were unknown, as they originated from various varieties of *V. vinifera* and *L. usitatissimum*. It is known that differences in maturation degrees, soil fertility, climate, and temperature, among other natural influences, impact the nutritional quality of fruits and seeds [42,43].

**Figure 2:**
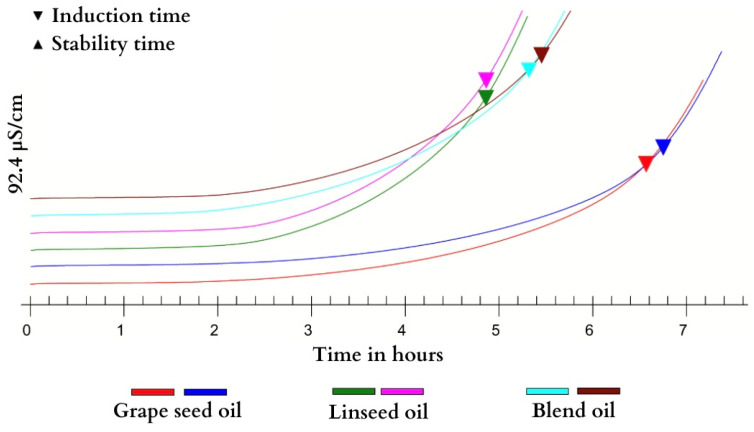
Oxidative stability of grape seed oil (G), linseed oil (L), and blend oil (GL) determined using the Rancimat method. The arrows indicate the formation of the graph originated by the analysis.

The Rancimat test, shown in Figure 2, indicates oxidative stability through the induction period (IP). It is a direct indication of the oxidation of vegetable oils during heating, with inversely proportional results; thus, the lower the content of PUFAs, the higher the IP value [27,44].

The grape seed oil (G1 and G2) obtained an IP of 6.66 h, the linseed oil (L1 and L2) obtained an IP of 4.86 h, and the blend oil (GL1 and GL2) obtained an IP of 5.39 h. The estimate of oxidative stability demonstrated by IP allowed us to detect of alterations caused by the increased temperature (range of 100 °C) and heat conductivity of the sample under controlled conditions and a constant airflow. 

Vegetable oils are susceptible to oxidative events because of their high content of unsaturated fatty acids, and polyunsaturated fatty acids are the most susceptible to loss during the first heating step due to their high number of double bonds [45]. G contained less PUFAs and more saturated fatty acids than GL and L (Table 3) [41]. Therefore, the oxidative stability (Figure 2) of G (G1 and G2) was higher than that of GL (GL1 and GL2) and L (L1 and L2). G demonstrated stability similar to O [46]. Such coincident behavior is due to the 12.20% content of total saturated fatty acids in O [20], with percentages similar to those of G (Table 3 and Figure 2). The oil with the second best thermal stability was GL (Figure 2) because it had a higher content of total saturated fatty acids and a lower presence of PUFAs than L (Table 3). GL presented an intermediate IP compared with that of G and L (Figure 2), with oxidative stability adequate for the behavior of pure oils.

L demonstrated a low oxidative stability (Figure 2) because of its high content of PUFAs. In particular, α-linolenic acid is more susceptible to degradation and has lower oxidative stability [44]. Nevertheless, some linseed cultivars present induction period (PI) values of 2.11–3.3 h [47], lower than what we found. Therefore, we suggest that linseed oil should not be utilized at high temperatures because of the loss of nutritional content and that it is best used as supplementation in capsules or added to cold preparations [45,46].

**Figure 3:**
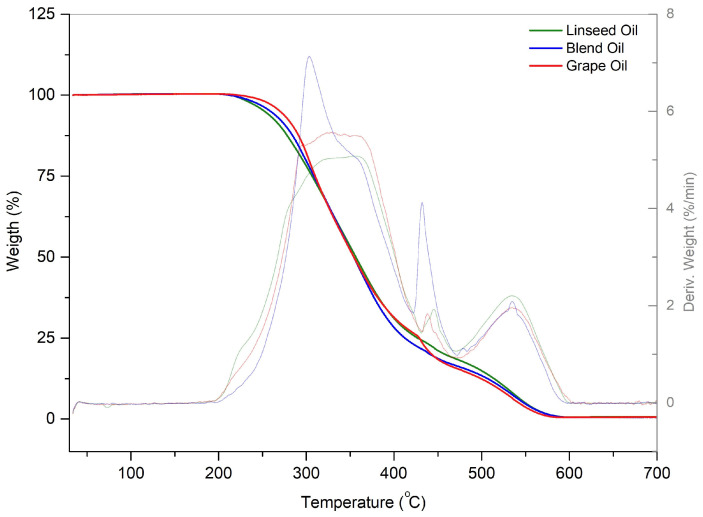
TG/DTG curves of grape seed oil (G), linseed oil (L), and blend oil (GL).

Thermogravimetry/Derivative Thermogravimetry (TG/DTG) and Differential Scanning Calorimetry (DSC) analyses show the thermal decomposition and transitions caused by a controlled variation in temperature [48]. The TG/DTG curves show the thermal decomposition process in G, L, and GL (Figure 3). In G, two steps of consecutive mass loss were observed at temperatures between 205 °C and 598 °C. For the correlation of the thermal decomposition profile, we calculated the mass losses in intervals. The first loss was 75.06%, starting at 210.92 °C until temperatures around 430.39 °C. The second loss was 24.72%, and occurred until the temperature of 594.64 °C. In L, we observed that the first decomposition step started at 207.70 °C until temperatures around 444.38 °C, with a mass loss of 78.01% and a second loss of 21.53% until the temperature of 590.69 °C. The first mass loss was observed in GL starting at 205.01 °C until 433.62 °C, with a loss of 78.85% and a final step of loss of 20.89% until 598.2 °C. The curves show similar thermal decomposition profiles; however, GL presented losses closer to those of L, overlapping the behavior of G.

Thus, no significant differences occurred in the mass losses. However, by comparing the decomposition profiles, we found that the first mass loss of G occurred at higher temperatures than that of the GL and L oils because of the higher content of saturated fatty acids (Table 3), which increased thermal stability and reduced mass loss [48,49]. GL presented a mass loss similar to that of L in both steps (Figure 3) because it had a higher presence of polyunsaturated fatty acids and a lower content of saturated fatty acids than G (Table 3). L from the province of Hebei, China showed low decomposition, with weight losses of 29.14% at 389 °C, 59.78% at 389 °C to 470 °C, and 11.08% at 470 °C to 600 °C [50], necessary to correlate the mass losses with the temperature intervals [51].

**Figure 4:**
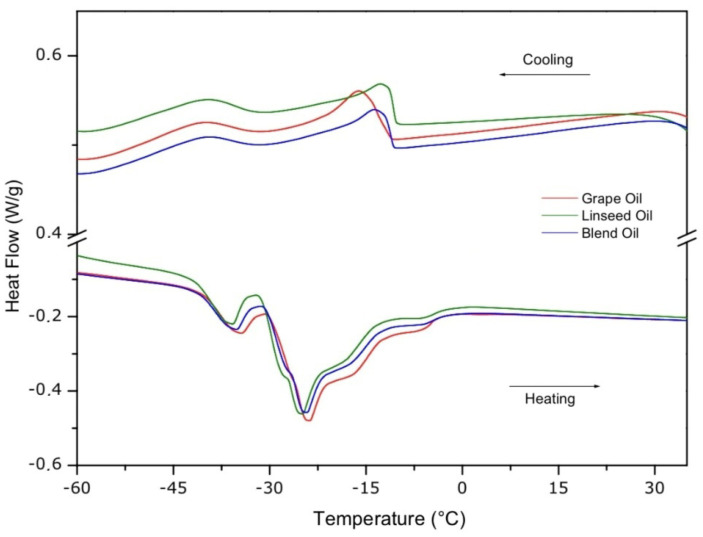
DSC curves of grape seed oil (G), linseed oil (L), and blend oil (GL).

The DSC curves show endothermic and exothermic peaks and the corresponding temperatures and absorbed or liberated energies during crystallization and fusion events in two steps (Figure 4). Our results show that GL has an intermediate crystallization temperature when compared with G and L.

In the cooling curve of G, we observed the first peak in the temperatures at −16.01 °C, corresponding to the crystallization temperature of saturated fatty acids, with an energy consumption of 4.864 J/g. Next, the second peak occurred at −42.36 °C, corresponding to the crystallization of unsaturated fatty acids, with an energy consumption of 3.152 J/g. In the linseed oil curve, the first crystallization peak occurred at −12.53 °C, with an energy consumption of 4.004 J/g for saturated fatty acids, and the second peak occurred at −41.53 °C, with an energy consumption of 3.549 J/g for unsaturated fatty acids. In the GL curve, the same events occurred at −13.66 °C, with an energy of 4305 J/g for saturated fatty acids, and the second peak occurred at a temperature of −41.68 °C, relative to the crystallization of unsaturated fatty acids, with an energy of 2.972 J/g.

Regarding the heating processes, we observed the corresponding temperatures and energies of the fusion peaks. In G, the first peak occurred at −40.72 °C, with a liberation of 5.370 J/g for unsaturated fatty acids, and the second peak occurred at a temperature of −28.37 °C, with a liberation of 38.74 J/g for saturated fatty acids. L showed the first peak at temperatures of −41.99 °C, with 6.330 J/g for unsaturated fatty acids, and the second peak occurred at −31.10 °C, with 40.22 J/g for saturated fatty acids. GL presented the first peak at a temperature of −41.17 °C, with 5.293 J/g for unsaturated fatty acids, and the second peak occurred at −28.89 °C, with 36.05 J/g for saturated fatty acids. The DSC curves allow us to conclude that GL had a reduced crystallization temperature and an increased fusion time compared with G, demonstrating similarities to the intervals of L.

The crystallization events alter the physical properties related to the oxidation process of vegetable oils, which are relevant quality parameters [52]. The heating curves serve to determine the functionality of these oils when incorporated into several food products. A safety interval for the manipulation of vegetable oils around 10 °C, close to the steps of crystallization and fusion, is recommended to conserve their physical–chemical properties [53]. The results for G, L, and GL did not indicate oxidative processes, demonstrating the integrity of their fatty acids (Table 3), as well as the quality and integrity of their composition (Table 4) [54]. Thus, under faster heating rates, L showed a crystallization profile with three exothermic peaks, with the highest peak at −63.54 °C and the two lower peaks at temperatures similar to those in our study of −40.11 °C and −15.37 °C [55]. Concerning the fusion, we found that L had characteristics similar to the peaks of −38.39 °C and −24.81 °C reported by Zhang et al. (2022) [55]. Other linseed cultivars show fusion peaks at −31.73 °C and −10.26 °C [56]. By comparing the fusion peaks of G and peanut oil, we found that the fusion temperature of G was lower, around −24.64 °C, with an enthalpy of 23.15 J/g [57]. These results are similar to the second fusion peak of G (Figure 4), correlated with the presence of antioxidants in the cultivated grape [14].

Antioxidant substances affect only the initial decomposition stage, preventing the formation of hydroperoxides [58]. The DSC curves of GL show accordance with the fatty acid profile (Table 3) and the analyses of identity and quality (Table 4), with values intermediate compared to those of G and L, with this pattern also observed in the thermal analyses.

**Figure 5:**
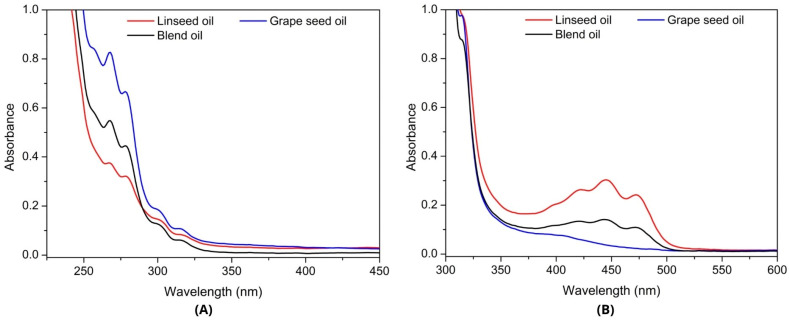
UV–vis absorption curves of grape seed oil (G), linseed oil (L), and blend oil (GL) (at (**A**) 5 g/dm^3^ and (**B**) 90 g/dm^3^ diluted in hexane).

**Figure 6:**
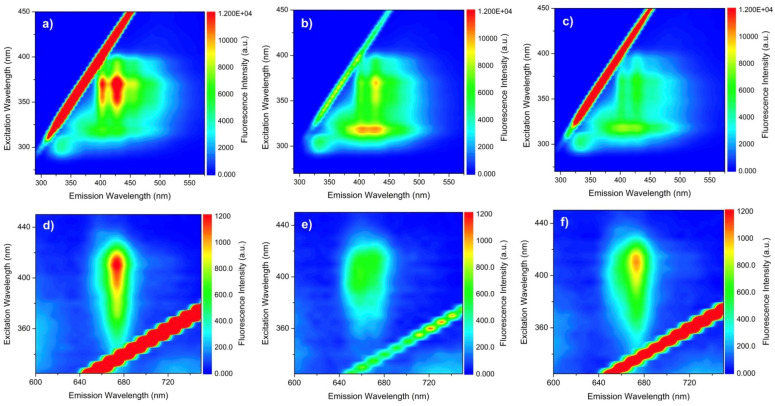
Excitation–emission maps of (**a**) linseed oil, (**b**) grape seed oil, and (**c**) blend oil in the 300–500 nm emission range and (**d**) linseed oil, (**e**) grape seed oil, and (**f**) blend oil in the 600–750 nm emission range.

Concerning the UV–vis absorption analyses, shown in Figure 5 and Figure 6, the results demonstrate a trend for the presence of carotenoids in L because of the higher value for this band, followed by the intermediate value of GL, which also presents results characteristic of the antioxidant group of carotenoids [59]; thus, they have a higher carotenoid content than G, as proven in the coloration analyses of each sampled oil (Table 5). 

The measured absorption spectra were between 5 g/dm^3^ and 90 g/dm^3^ (Figure 5A,B). Different concentrations provided evidence of the UV–vis absorption bands of G, L, and GL. Regarding absorbance in the 250–290 nm band detected at 5 g/dm^3^ (Figure 5A), G demonstrated the highest value, followed by GL with intermediate absorbance behavior.

The 400–500 nm band was detected for the 90 g/dm^3^ concentration of G, L and GL (Figure 5B). L presented the highest value in this band, followed by GL with an intermediate value; thus, the presence of carotenoids in L and GL was superior to that in G. 

Concerning the fluorescence analysis of the vegetable oils (Figure 6), it was possible to note that fluorescence centered at 330 nm with excitation at 300 nm (first band), demonstrating the highest intensity for L (a), followed by GL (c). In the emission region of 350 to 470 nm with excitation between 300 and 400 nm (second band), G (b), L, and GL presented similar emission profiles, with, however, different fluorescence intensities.

It is possible to observe fluorescence centered at 330 nm with excitation at 300 nm (first band), which is associated with the emission of tocopherols and polyphenols [60]. The emission region of 350 to 470 nm with excitation between 300 and 400 nm can be attributed to the emissions of chlorophyll and methyl esters [60,61], with variations in the higher-intensity band due to differences in the compositions of antioxidants of the fatty acids of the oils. The second fluorescence region with a lower intensity was detected at 330 nm with excitation at 300 nm, characteristic of chlorophyll emission [62]. The highest presence of this compound was observed in G, followed by in GL (Table 5). The phenolic compounds protect vegetable oils against the free radicals involved in lipid peroxidation and act directly on oxidative reactions, impeding the action of pro-oxidant agents [63]. 

A second fluorescence region on had a lower intensity than the first region, presenting a maximum emission intensity at around 675 nm when excited at 410 nm. The fluorescence of L in this region was more intense than that of G, while GL demonstrated a fluorescence intensity intermediate compared to that of G and L.

Other analyses must be carried out to complement the identification and quantification of phenolic compounds, such as the applications of mass spectrometry systems (MS, MS/MS), use of mass detectors, liquid chromatography coupled to mass spectrometry (LC-MS/MS), high-performance liquid chromatography (HPLC), paper chromatography and thin layer chromatography (TLC), high-speed countercurrent chromatography (HSCCC), capillary electrophoresis (CE), and supercritical fluid chromatography (SFC). In addition, high-resolution systems such as time of flight (TOF-MS) and ion trap-MS focus on unknown compounds [64,65].

Regarding the colorimetric parameters of G, L, and GL (Table 5), G and GL showed a darker color (L*) than L. Concerning color saturation (C*), G demonstrated the lowest concentration of pigments, and L was similar to GL, presenting more intense colors. Regarding a* (red axe (+)/green (−)), G presented a greenish color; L presented an a* positive result, indicating coloration with orange tones; and GL stayed a yellowish color. For b* (yellow axe (+)/blue (−)), all results were positive, indicating yellow tones for all oils, with G showing less yellow than L and GL.

When the Hue angle (°) was observed, an indicator of tone, G exhibited a greyish yellow–green color, L showed a yellow color with orange tones, and GL demonstrated a bright yellow color.

The colorimetric parameters of G indicated greyish coloration because of the low content of yellow and orange pigments (Table 5), but L and GL had a significant presence of these pigments of bright colors. Yellow to reddish pigments indicate carotenoids, which were mainly observed in L and GL, with β-carotene being the most abundant, as shown in Table 5 and Table 6. The presence of β-carotene in vegetable oils benefits utilization and conservation, as it counteracts the reactive species of oxygen and free radicals [66].

The quantification of carotenoids in the oils was directly related to the emission–excitation (Figure 6), coloration (Table 5), quality, and identity analyses (Table 4), confirming the results [21]. A comparison of the total carotenoid contents of G, L, and GL (Table 6) showed that linseed oil had the highest carotenoid levels, followed by GL and G.

The high CV values are caused by the easy degradation of carotenoids under exposure to light and air oxygen, as well as solvent evaporation during the absorbance determination.

The carotenoids associated with the chlorophyll in plants are punctuated by values of “a”, a blue-greenish color, and “b”, a greenish-yellow color (Table 6) [67]. Carotenoids are liposoluble compounds of vegetable oils [63]. Thus, the coloration (Table 5) confirms the presence of β-carotene (Table 6) mainly in L, followed by in GL. β-carotene is the primary precursor of vitamin A [67].

Nonetheless, G, even with low concentrations of carotenoids, contains polyphenols and vitamin E, which enhance wound healing and reduce systemic inflammation [68]. Compared with other vegetable oils, G possesses a higher content of vitamin E isomers, such as tocotrienol, known for their antioxidant, anti-inflammatory, and antitumor properties, and this distinguishes the oil in terms of antioxidant activity [69]. Furthermore, the easy degradation of carotenoids under exposure to light, oxygen, and solvent evaporation during oil analysis and extraction from seeds can alter their quantification [66,70]. Variations in carotenoid content are influenced by the genetic characteristics of the plant variety, environmental conditions, maturation degree, and oil extraction method [71].

Likewise, flavonoids, phenolic acids, and tocopherols are unsaponifiable products with anti-inflammatory properties and regenerative effects [49], isolated from G and L, which demonstrate anti-inflammatory properties and regenerative effects [49]. Additionally, we found that GL has intermediate results compared to both crude G and L, with possible beneficial actions to health for attributions specific to each oil, demonstrating a synergic effect.

Concerning the animal experimentation, while both linseed oil and grape seed oil have garnered scientific attention and commercial viability, their prominence in research emerged primarily in the last century. In contrast, olive oil has a rich historical tradition, having been consumed and studied for centuries within Mediterranean cultures and globally [15,31].

The health benefits, historical use, scientific support, culinary versatility, and global popularity of olive oil contributed to the decision to use this oil in the control group supplemented with oil (o). Olive oil is rich in polyphenols, particularly hydroxytyrosol and oleuropein, with antioxidant properties. These compounds act by scavenging free radicals and mitigating oxidative damage to cells, exerting anti-inflammatory effects through the modulation of inflammatory pathways associated with cardiovascular conditions and cancer [72].

Concerning the total weight gain of animals, as presented in Table 7, the experimental groups did not show significant differences in total weight gain between the start of the experiment (*p* = 0.877) and the experiment end (*p* = 0.290). After eleven weeks of treatment, the control group © exhibited a significantly higher weight gain than the groups that received the grape seed oil (G1 and G2) and linseed oil at a dosage of 2000 mg/kg/day (L2) (*p* < 0.05). The GL group showed a lower total weight gain than the C and O groups (*p* < 0.001 and *p* < 0.05, respectively).

Concerning total feed consumption in the 11-week experiment (Table 7), which refers to the total feed intake of each mouse for the experimental period of 11 weeks, C showed more consumption than the O, L2, G1, and G2 groups (*p* < 0.05). The L1 group also showed more consumption than the O and G2 groups (*p* < 0.05), and G1 and GL showed more consumption than G2 (*p* < 0.05). 

After the eleven-week experimental period, we evaluated the feed effectiveness coefficient (FEC) and weight gain per caloric consumption (GWGCI) according to the intake of each group (as detailed in Table 7). Notably, the GL group exhibited significantly lower weight gain (*p* < 0.05) than both C and L1 groups, whether measured per gram of consumed feed or in terms of total caloric consumption. Total feed consumption was the lowest in G2 and O groups (also indicated in Table 7), and total consumption was the lowest in G2 and O groups. Nevertheless, GL had higher total consumption than G2 but lower final weight gain when compared to C and L1.

It is known that supplementation with vegetable oils does not influence calorie intake. The oil dosages in ml ranged from 0.03 ± 0.02 mL/day to 0.05 ± 0.03 mL/day, with weekly adjustment according to weight evolution, so the additional calories from the supplement varied from 0.27 ± 0.1 kcal/day to 0.44 ± 0.2 kcal/day, according to the dosages of 1.000 mg/ kg/day and 2.000 mg/kg/day.

Our results confirm that vegetable oils supplemented with nutritional and effective functional properties can act on an organism as nutraceuticals [13]. Linoleic acid (C18:2) and α-linolenic fatty acid (C18:3) induce the lengthening of carbon chains (more than 22 C atoms) and are described as “essential fatty acids” because they must be in the diet [73,74]. Thus, the supplementation of GL, composed of a 1:1 (*v*/*v*) ratio of G and L, ratifies the significant presence of both fatty acids (Table 3) and shows the beneficial utilization of macronutrients in the ingested feed, resulting in the total weight gain, CEA, and CGPCC being lower than in the other groups.

Compared with L and GL, the higher satiety and lower weight gain caused by the supplementation of G at a dosage of 2000 mg/kg/day (G2) can be justified by the eminent exposure of the animals to quantities of monounsaturated fatty acids (Table 3). Additionally, the higher total consumption of group C can be attributed to the satiety and caloric percentage of the distilled water supplementation being lower than those of the vegetable oil supplementation [75].

**Figure 7:**
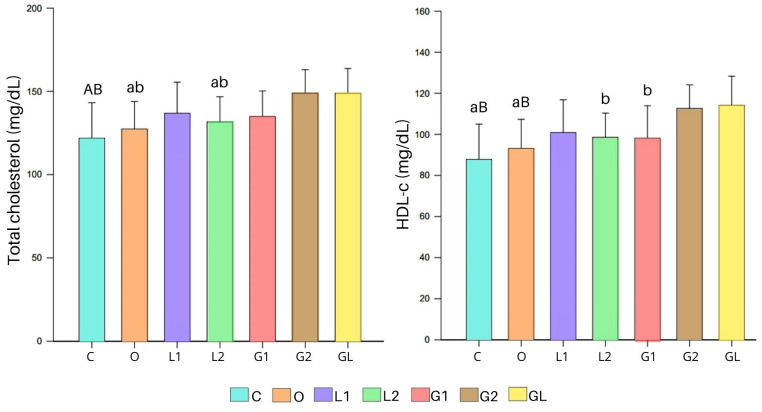
Serum parameters of total cholesterol and HDL-c at the end of the 11-week experiment. C: Control group (distilled water at dose of 1000 mg/kg/animal); O: Extra virgin olive oil group (1000 mg/kg/animal); L1: Golden linseed oil group (1000 mg/kg/animal); L2: Golden linseed oil group (2000 mg/kg/animal); G1: Grape seed oil group (1000 mg/kg/animal); G2: Grape seed oil group (2000 mg/kg/animal); GL: Blend oil (2000 mg/kg/animal). Mean ± standard deviation from the mean. Different letters indicate significant differences in relation to G2 (*p* < 0.05: a; *p* < 0.001: A) and GL (*p* < 0.05: b; *p* < 0.001: B), determined via one-way ANOVA with Tukey’s post-test.

Concerning serum parameters, shown in Figure 7, fastening glycemia did not show significant differences (*p* = 0.153) between the experimental groups. Similarly, triglyceride levels did not show differences between the groups (*p* = 0.052); however, L1 had the highest values. Regarding total cholesterol, group C showed the lowest value compared with G2 and GL (*p* < 0.001), and O and L2 also had lower total cholesterol than G2 and GL (*p* < 0.05) (Figure 7). This demonstrates that, in C, O and L2, fewer changes occurred in the transport of cholesterol and triglycerides and the reverse transport of cholesterol to the liver, where they are metabolized [76]. 

HDL-c levels were higher in GL and G2 than in C (*p* < 0.001) and O (GL: *p* < 0.001; G2: *p* < 0.05); additionally, group GL exhibited higher HDL-c levels than G1 (*p* < 0.05) (Figure 7). Demonstrating that, in C and O, fewer changes occurred in the transport of cholesterol and triglycerides and the reverse transport of cholesterol to the liver, where they are me-tabolized [76]. Concerning LDL-c, VLDL-c, and non-HDL cholesterol, we did not observe significant differences between the groups (*p* = 0.531, *p* = 0.052, and *p* = 0.082, respectively).

The experimental groups, mainly G2 and GL, despite not receiving a significant amount of calories from the oil, showed the lowest consumption and weight gain, which can be attributed to the greater satiety and sensation of food intake from the oil [75]. Furthermore, the supplementation of oils may have impacted cholesterol levels, leading to a different metabolic response related to their lipid profile compared with the control group [77]. The increase in HDL-c cholesterol levels, referred to as “good cholesterol”, mainly observed in G and GL, can be attributed to the composition of fatty acids, the presence of antioxidant compounds, and the unique physicochemical characteristics of the supplemented oils contributing to this favorable outcome [78].

Group C, which did not receive oil supplementation, showed the highest food consumption and a significant total weight gain (Table 3). However, this group showed the lowest total cholesterol levels, without changes in the blood lipid profile [76,78].

The L2 and O groups showed significantly lower total cholesterol values than the G2 and GL groups. Although the olive oil supplementation at 1000 mg/kg/day did not influence on weight control, it did affect total cholesterol levels. The monounsaturated fatty acids (MUFAs) present in olive oil play a crucial role in increasing nitric oxide production, impacting endothelial function [79]. Although an increase in HDL-c was not demonstrated in this study, Millman et al. (2021) [72] found that olive oil consumption reduces low-density lipoprotein (LDL-c) cholesterol levels while increasing high-density lipoprotein (HDL-c) cholesterol levels.

Additionally, the significant reduction in cholesterol in the L2 group could have had cardio-protective, antitumoral, and anti-inflammatory effects via the metabolic action of α-linolenic fatty acid (C18:3), with this group showing a trend of reduced levels of triglycerides and LDL-c [80,81]. This predisposition was not noted for the L1 group.

In this study, the G2 and GL groups exhibited higher levels of high-density lipoprotein cholesterol (HDL-c), while the HDL-c of GL surpassed that of G1 (as shown in Figure 7). Thus, in addition to promoting better weight maintenance, GL significantly elevated HDL-c levels. These findings demonstrate that improved treatments at the doses of 2000 mg/kg/day administered to the G2 and GL groups exhibited anti-inflammatory characteristics, leading to weight control and increased plasma HDL-c levels [79].

The GL dosage suggests that the relative proportions of the fatty acids in the grape seed oil and linseed oil complement each other, resulting in an antioxidant effect. Combined, these oils may offer enhanced health benefits due to their unique fatty acid profiles [79,80]. While grape seed oil is rich in linoleic acid, linseed oil provides a significant amount of alpha-linolenic acid (ALA), both contributing to overall health and oxidative protection and increase HDL-c. The synergy between these oils underscores their potential as nutraceuticals [80]. In this study, it was observed that grape seed oil (G) at the highest dosage of 2000 mg/kg/day and GL at the same dosage were the most effective supplements for increasing high-density lipoprotein cholesterol (HDL-c) levels and for causing the lowest weight gain in the studied animals. Interestingly, the high total cholesterol levels in these groups did not suggest any disease-related changes.

**Figure 8:**
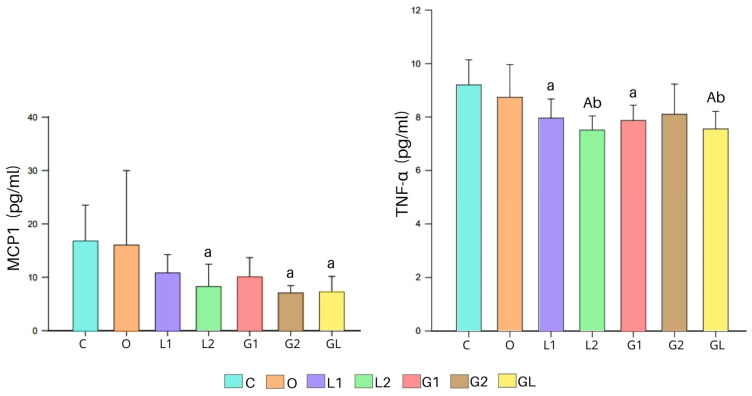
Concentrations of MCP1 and TNF-α cytokines at the end of 11-week experiment. C: Control group (distilled water at dose of 1000 mg/kg/animal); O: Extra virgin olive oil group (1000 mg/kg/animal); L1: Golden linseed oil group (1000 mg/kg/animal); L2: Golden linseed oil group (2000 mg/kg/animal); G1: Grape seed oil group (1000 mg/kg/animal); G2: Grape seed oil group (2000 mg/kg/animal); GL: Blend oil (2000 mg/kg/animal). Mean ± standard deviation from the mean. Different letters in the same bar indicate significant differences in relation to C (*p* < 0.05: a; *p* < 0.001: A) and O (*p* < 0.05: b), determined via one-way ANOVA with Tukey’s post-test. MCP1 determined via Kruskal–Wallis test followed by Dunn’s test (Kruskal–Wallis/Dunns).

The concentrations of cytokines measured in the blood serum of the experimental groups, shown in the Figure 8, showed lower MCP1 levels in L2, G2, and GL than in C (*p* < 0.05). Regarding IL-6, we did not detect significant differences between the groups (*p* = 0.199). The TNF-α values were lower in L2, GL (*p* < 0.001), L1, and G1 (*p* < 0.05) than in C (Figure 8). The L2 and GL groups also presented lower levels than O (Figure 8).

The PAI-1 values did not show differences between the groups (*p* = 0.697). Additionally, regarding the hormones of insulin, leptin, and resistin, we did not detect a significant difference between the groups (*p* = 0.165, *p* = 0.121, and *p* = 0.091, respectively), with the lowest insulin levels in group L1, lowest leptin values in group O, and lowest resistin levels in group C.

The L2, G2, and GL groups exhibited reduced levels of pro-inflammatory cytokines (MCP-1 and TNF-α), highlighting the anti-inflammatory action of their supplementation. This effect can be partly attributed to the fatty acid (FA) profiles of the oils, particularly the presence of linoleic acid (LA) at percentages of 52.4%, 14.32%, and 42.53% for L2, G2, and GL, respectively.

LA, a common omega-6 fatty acid, undergoes metabolism to other omega-6 polyunsaturated fatty acids (PUFAs). Among these, arachidonic acid (ARA) significantly contributes to the composition of membrane phospholipids in cells involved in inflammation. ARA serves as a precursor to pro-inflammatory mediators, including prostaglandins and leukotrienes, which are targeted by anti-inflammatory pharmaceuticals for inflammation control [82].

However, an excessive omega-6 intake can intensify inflammatory processes. Conditions such as hypertension, increased pain in arthritis cases, and even inflammatory bowel diseases may result from an imbalance. Interestingly, studies in healthy adults have shown that increased LA consumption does not necessarily increase the concentrations of many inflammatory markers. Indeed, epidemiological evidence has suggested that LA might be associated with reduced inflammation [83].

Ferrucci and colleagues observed that total n-6 PUFA plasma concentrations were inversely associated with serum CRP, IL-6, IL-6r, IL-1ra, and TNF in a cross-sectional analysis of 1123 Italian adults [80]. Another study found an inverse and/or no association between plasma or dietary LA and a variety of markers of chronic inflammation, and other explanations for the proinflammatory LA hypothesis have been offered by several investigators [83].

In contrast, the dietary supplementation had beneficial effects on the L1, L2, GL, and G1 groups, as evidenced by the lower tumor necrosis factor-alpha (TNF-α) levels, shown in Figure 8. This reduction indicates a possible decreased activation of the NF-κB pathway and an improved endothelial cell integrity due to a reduced pro-inflammatory cytokine expression [83,84] that correspond to decreased TNF binding to its receptor TNFR1, causing lesser activation of inflammatory genes [85]. However, in the C and O groups, the results, potentially linked to the greater weight gain during the 11-week experiment and differences in adipocyte size, may indicate low-grade chronic inflammation [84,86]

**Figure 9:**
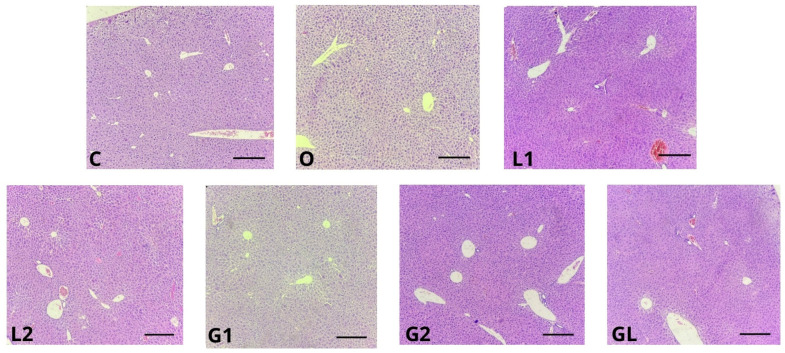
Histology of animal hepatocytes 100×. C: Control group (distilled water at dose of 1000 mg/kg/animal); O: Extra virgin olive oil group (1000 mg/kg/animal); L1: Golden linseed oil group (1000 mg/kg/animal); L2: Golden linseed oil group (2000 mg/kg/animal); G1: Grape seed oil group (1000 mg/kg/animal); G2: Grape seed oil group (2000 mg/kg/animal); GL: Blend oil (2000 mg/kg/animal). Mean ± standard deviation from the mean. Kruskal–Wallis/Dunn’s tests. Scale bar = 100 μm; original magnification × 100.

Concerning the histology of the hepatic tissue (Table 8), all groups presented an absence of steatosis (<5%) and microvesicle steatosis (both *p* = 1.000). Regarding lobular inflammation, we did not find significant differences between the groups (*p* = 0.528). For the analysis of ballooning hepatocytes (Table 8 and Figure 9), G2 exhibited a prevalence of many cells compared with the C, L1, and GL groups (*p* = 0.008). However, all groups presented an absence of Mallory’s hyaline (*p* = 0.421), apoptosis (*p* = 0.143), and glycogenated nuclei (*p* = 0.249). 

Furthermore, steatosis (fatty liver) was not detected in any group (Table 8 and Figure 9). This finding suggests that, in both the control group receiving olive oil (O) and the experimental groups supplemented with grape seed oil (G1 and G2), linseed oil (L1 and L2), and blend oil (GL), liver damage was not induced. Neither group C (control) nor the experimental animals exhibited microvesicular steatosis or lobular inflammation. 

In our study, we did not observe notable swollen hepatocytes with cytoplasm fat droplets, a characteristic feature of hepatocellular ballooning [86]. While significant differences were detected in this analysis (as shown in Table 8 and Figure 9), other hepatic parameters such as the presence of Mallory’s hyaline, apoptosis, and glycogenated nuclei remained unaltered. Notably, the identification of ballooned hepatocytes in hematoxylin and eosin-stained slides has limitations, including sampling variability and inconsistent lesion distributions among observers [85,87].

The dietary supplementations did not significantly impact the hepatic function. However, certain diet components can positively influence metabolic and hepatic health. For instance, vitamin E, known for its antioxidant properties, and polyphenols, which reduce oxidative stress and liver inflammation, may offer hepatoprotective effects [76,88].

**Figure 10:**
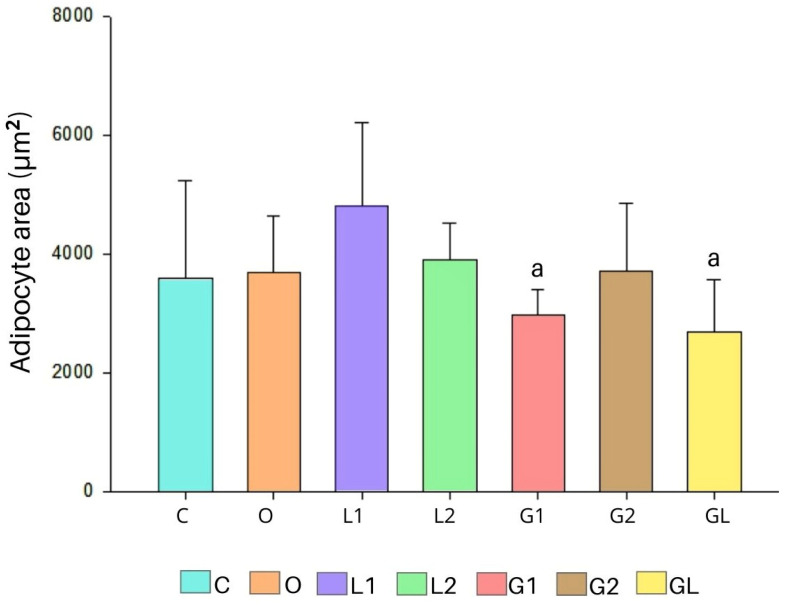
Adipocyte area (μm^2^) of animals at the end of the 11-week experiment. C: Control group (distilled water at dose of 1000 mg/kg/animal); O: extra virgin olive oil group (1000 mg/kg/animal); L1: Golden linseed oil group (1000 mg/kg/animal); L2: Golden linseed oil group (2000 mg/kg/animal); G1: Grape seed oil group (1000 mg/kg/animal); G2: Grape seed oil group (2000 mg/kg/animal); GL: Blend oil (2000 mg/kg/animal). Mean ± standard deviation from the mean. Different letters indicate significant differences in relation to L1 (*p* < 0.05: a). Kruskal–Wallis/Dunn’s tests.

**Figure 11:**
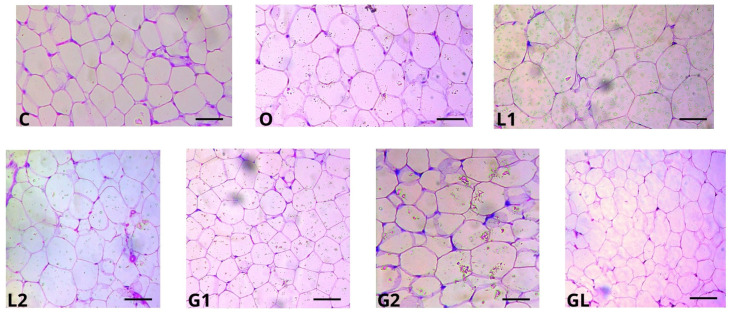
Histopathological analyses of the epididymal adipose tissue of the animals at the end of the 11-week experiment. G: Control group (distilled water at dose of 1000 mg/kg/animal); O: Extra virgin olive oil group (1000 mg/kg/animal); L1: Golden linseed oil group (1000 mg/kg/animal); L2: Golden linseed oil group (2000 mg/kg/animal); G1: Grape seed oil group (1000 mg/kg/animal); G2: Grape seed oil group (2000 mg/kg/animal); GL: Blend oil (2000 mg/kg/animal). Histopathological analyses of the adipose tissue via hematoxylin and eosin (H&E); 20× magnification; bar scale: 100 μm.

By assessing the adipocyte area of the epidydimal adipose tissue, Figure 10, we found that the G1 and GL groups presented lower areas than the L1 group (*p* < 0.05) (Figure 11).

In our investigation of epididymal adipose tissue (as illustrated in Figure 10 and Figure 11), we observed that both the G1 and GL groups exhibited smaller adipocyte areas than the L1 group. Adipose tissue, functioning as an energy reservoir, plays a crucial role in maintaining metabolic homeostasis by storing excess energy derived from the diet. However, when there is a positive energy balance, this tissue undergoes reorganization, increasing both adipocyte number and size. Notably, the L1 group, which gained more weight, displayed a larger adipose tissue area. Furthermore, we found that L1 also produced lower levels of TNF levels (tumor necrosis factor) and MCP-1 (monocyte chemoattractant protein-1) compared with the other groups. These findings underscore the intricate relationship between adipose tissue expansion, cytokine production, and weight gain, with larger adipocytes potentially accumulating more fat due to cell hypertrophy.

In our assessment of epididymal adipose tissue (as depicted in Figure 10 and Figure 11), we observed that the G1 and GL groups exhibited smaller adipocyte areas than L1. Adi-pose tissue serves as an energy reservoir, maintaining homeostasis by storing excess energy from the diet. When there is a positive energy balance, this tissue undergoes reorganization, leading to an increase in both adipocyte number and size. Larger adipocytes can grow fatter due to cell hypertrophy and higher weight gain in the L1 group [89,90].

Di Pietro et al. (2023) [13] emphasized the relevance of grape seed oil as a therapeutic compound with active properties for health, pointing out several in vitro and in vivo studies that show the modulation of the expression of antioxidant enzymes, anti-inflammatory and anti-atherosclerotic effects, and protection against cell oxidative damage. Thus, our results of the excellent therapeutic fraction of 2.000 mg/kg/day of G2 and its combination with L in GL agreed with the results of that report [13].

The response to oxidative stress and the improved antioxidant system provided by the blend oil are observed in the reduced size of adipocytes. Hence, the beneficial effects of the moderate consumption of vegetable oils, as well as their combination, can help reduce obesity given their physical–chemical quality, the presence of bioactive compounds, and their antioxidant and anti-inflammatory properties [21,30].

Grape seeds offer significant health benefits due to their high antioxidant potential. These benefits include protection against oxidative damage, anti-diabetic effects, cholesterol regulation, and anti-platelet properties [13]. Grape seed oil, rich in natural antioxidants, finds application in the food industry, where it extends the shelf life of various food products. This natural alternative is particularly valuable given the potential carcinogenic and toxic effects associated with synthetic antioxidants, such as tertiary butylhydroquinone (TBHQ), butylated hydroxyanisole (BHA), and butylated hydroxytoluene (BHT). Notably, grape seeds have been used as food additives in Japan [91].

Additionally, grape seed extracts find application in cosmetic formulations for their anti-aging properties. These extracts, abundant in proanthocyanidins, exhibit robust free radical scavenging capabilities. Recent studies have explored topical applications of grape seed extracts [92,93]

Linseeds also offer specific nutritional advantages. Notably, they are rich in omega-3 fatty acids and contain extraordinarily high levels of alpha-linolenic acid (ALA). Additionally, linseeds provide a significant lignan content and mucilage gums, making them a desirable commodity in the food industry [94]. 

This study aimed to comprehensively evaluate the combination of grape seed oil and linseed oil. Analytical techniques provided insights into the oils’ physical behavior, which impacts storage stability and use in supplementation. Gas chromatography–mass spectrometry verified the oil blend composition, confirming the specific fatty acids and bioactive compounds, with carotenoids and color observations complementing these analyses.

By intentionally utilizing an in vivo experimental model with eutrophic feeding, we specifically investigated the physiological effects of the oil mixture. Despite the widespread use of grape seed oil and linseed oil as dietary supplements, robust evidence supporting the physiological benefits of their joint use remains elusive. Our findings indicate that this combination may indeed impact lipid profiles, indicators of inflammation, and antioxidant status.

## 4. Conclusions

The predominant polyunsaturated fatty acid in the grape seed oil and blend oil is linoleic acid (C18:2), while that in the linseed oil is alfa-linolenic acid (C18:3). In all oils, oleic acid (C18:1) is the dominant monounsaturated fatty acid, and palmitic (C16:0) was the leading saturated fatty acid. The physical–chemical indices of the studied oils are within the recommended ranges, indicating the absence of oxidation. Oxidative stability and thermal analyses (TGA/DTA and DSC) revealed similar behaviors, and optical analyses showed color variations caused by pigments (carotenoids).

The grape seed oil at a dosage of 2000 mg/kg/day (G2) promotes lower food consumption, and the oil blend (GL) causes a lower weight gain per gram of consumed feed and total caloric consumption. Both oils induce high lipoprotein HDL-c levels, while linseed oil (2000 mg/kg/day) (L2) reduce total cholesterol levels. Furthermore, L2, G2, and GL show reduced levels of pro-inflammatory cytokines (MCP-1 and TNF-α), highlighting the anti-inflammatory effects of their supplementation; G2 and GL exhibit the smallest adipocyte areas.

Future studies should be conducted on the application of grape seed, linseed, and blend oils, as their nutraceutical properties have potential uses in the food, pharmaceutical, and cosmetic industries.

## Figures and Tables

**Table 1:** Determination of experimental groups, supplementation, and dosages.

Groups	Supplementation	Dosages (mg/kg/animal)
Control (C)	Distilled water	1000
Olive oil (O)	Olive oil	1000
Linseed oil (L1)	Golden linseed oil	1000
Linseed oil (L2)	Golden linseed oil	2000
Grape seed oil (G1)	Grape seed oil	1000
Grape seed oil (G2)	Grape seed oil	2000
Blend oil (GL) *	Blend oil 1:1 (*v*/*v*) *	2000

* Blend oil: Grape seed oil + Linseed oil 1:1 (*v*/*v*).

**Table 2:** Composition of the commercial normocaloric feed (g/kg feed).

Ingredients (g/kg)	Nuvital^®^
Starch	725.67
Casein (≥82% protein)	40.00
DL-methionine	100.00
Soy oil	40.00
Cellulose	100.00
Mineral mix **	35.00
Vitamin mix **	10.00
L-cystine	1.80
Choline bitartrate	2.50
Tertbutyl hydroquinone	0.008
Energy (kcal/kg)	4360.00
Carbohydrates (%)	75.75%
Proteins (%)	16.00%
Lipids (%)	8.25%
Calories/g diet	4.36

** Mix of vitamins and minerals according to the manufacturer.

**Table 3:** Fatty acid profile (%) of grape seed oil, linseed oil, and blend oil.

Fatty Acids	Grape Seed Oil (G) (%)	Linseed Oil (L) (%)	Blend Oil (GL) (%)
Myristic acid (C14:0)	0.07 ± 0.01	0.1 ± 0.01	0.06 ± 0.02
Palmitic acid (C16:0)	9.2 ± 0.1	5.8 ± 0.05	8.47 ± 0.07
Heptadecanoic acid (C17:0)	0.07 ± 0.06	0.1 ± 0.02	0.07 ± 0.01
Stearic acid (C18:0)	3.66 ± 0.02	3.9 ± 0.06	3.91 ± 0.07
Σ SATURATED	13 ± 0.18	9.9 ± 0.15	12.45 ± 0.17
Palmitoleic acid (C16:1ω7)	0.07 ± 0.01	0.1 ± 0.01	0.07 ± 0.01
Oleic acid (C18:1ω-9)	27.94 ± 0.8	18.31 ± 1.0	25.57 ± 0.07
Elaidic acid (C18:1*trans*-9)	1.12 ± 0.05	0.1 ± 0.04	1.0 9± 0.03
Gadoleic acid (C20:1ω-9)	0.03 ± 0.01	0.2 ± 0.01	0.2 ± 0.01
Σ MONOUNSATURATED	29.16 ± 0.9	18.71 ± 1.06	26.93 ± 0.12
Linoleic acid (C18:2ω-6)	52.40 ± 1.34	14.32 ± 1.00	42.53 ± 0.2
Gamma-Linolenic acid (C18:3ω-6)	0.14 ± 0.01	0.2 ± 0.01	0.16 ± 0.01
Alpha-linolenic acid (C18:3ω-3)	0.37 ± 0.04	53.30 ± 0.03	16.76 ± 0.06
Cis-5,8,11,14,17-Eicosapentaenoic acid (C20:5ω-3)	0.04 ± 0.01	3 ± 0.01	0.1 ± 0.03
Arachidonic acid (C20:4ω-6)	0.5 ± 0.04	0.1 ± 0.02	0.38 ± 0.01
Σ POLY-UNSATURATED	53.45 ± 1.4	70,92 ± 1.07	59.93 ± 0.31
Σ TOTAL UNSATURATED FATTY ACIDS	82.61%	89.63%	86.86%
Atherogenicity index	0.11	0.7	0.1
Thrombogenicity index	0.3	0.05	0.14

Values expressed as mean ± standard deviation.

**Table 4:** Quality and identity scores of grape seed oil, linseed oil, and blend oil 1:1 (*v*/*v*).

Indices	Grape Seed Oil (G)	Linseed Oil (L)	Blend Oil (GL)
Acidity (mg KOH/g)	1.2	2.8	1.3
Peroxide (mEqO_2_/kg)	6.5	2.0	4.6
Refraction (40 °C)	1.470	1.477	1.473
Iodide (g I_2_/100 g)	122.71	175	128.59
Saponification (mg KOH/g)	192	196	192
Relative density (mg/mL)	0.910	0.927	0.918

Values expressed as the mean ± standard deviation of the mean.

**Table 5:** Colorimetric parameters of grape seed oil, linseed oil, and blend oil.

Parameters	Grape Seed Oil (G) (%)	Linseed Oil (L) (%)	Blend Oil (GL) (%)
L*	50.32	38.42	47.15
C*	22.31	38.70	39.27
*Hue* (°)	−70.23	82.40	88.72
a*	−7.55	5.12	0.88
b*	20.99	38.36	39.26

* The means were determined from triplicates. Values are expressed as the mean ± standard deviation of the mean.

**Table 6:** Carotenoid content in grape seed oil, linseed oil, and blend oil.

Oil	Carotenoids (µg/g)	CV *
Grape seed oil (G)	1.16 ± 0.15 ^c^	0.13
Linseed oil (L)	13.67 ± 1.74 ^a^	0.13
Blend oil (GL)	6.23± 0.54 ^b^	0.88

* CV: coefficient of variation. The means were determined from triplicates. Values are expressed as the mean ± standard deviation. The same letters in the same column mean no significant difference at the 5% level.

**Table 7:** Behavioral parameters of weight, food consumption, FEC, and CWGCI of animals after 11 weeks of the experiment.

Parameters	C	O	L1	L2	G1	G2	GL
Starting weight (g)	35.94 ± 4.34	35.50 ± 3.54	35.33 ± 3.16	36.50 ± 4.46	35.12 ± 2.98	36.65 ± 3.35	36.83 ± 4.14
Final weight (g)	43.31 ± 6.16	40.83 ± 5.49	41.39 ± 4.65	40.06 ± 5.68	39.06 ± 5.46	40.47 ± 4.06	39.44 ± 4.49
Total weight gain (g)	7.37 ±3.48	5.33 ± 3.07	6.06 ± 3.64	3.56 ± 3.09 ^a^	2.94 ± 3.15 ^a^	3.82 ± 2.45 ^a^	2.61 ± 2.93 ^Ab^
Total consumption (g) ^1^	459.67 ± 68.96	346.78 ± 29.27 ^ac^	398.22 ± 36.84	355.61 ± 45.18 ^a^	376.47 ± 34.49 ^a^	327.59 ± 18.58 ^acde^	383.11 ± 40.70
FEC	1.65 × 10^−2^ ± 0.8 × 10^−2^	1.49 × 10^−2^ ± 0.7 × 10^−2^	1.55 × 10^−2^ ± 0.8 × 10^−2^	1.05 × 10^−2^ ± 0.8 × 10^−2^	0.8 × 10^−2^ ± 0.7 × 10^−2^	1.18 × 10^−2^ ± 0.7 × 10^−2^	0.7 × 10^−2^ ± 0.7 × 10^−2 ac^
CWGCI	0.38 × 10^−2^ ± 0.1 × 10^−2^	0.34 × 10^−2^ ± 0.1 × 10^−2^	0.35 × 10^−2^ ± 0.2 × 10^−2^	0.24 × 10^−2^ ± 0.1 × 10^−2^	0.19 × 10^−2^ ± 0.1 × 10^−2 a^	0.27 × 10^−2^ ± 0.1 × 10^−2^	0.16 × 10^−2^ ± 0.1 × 10^−2 ac^

FEC: feed effectiveness coefficient; CWGCI: weight gain per caloric consumption index; C: control group (distilled water at dose of 1000 mg/kg/animal); O: extra virgin olive oil group (1000 mg/kg/animal); L1: Golden linseed oil group (1000 mg/kg/animal); L2: Golden linseed oil group (2000 mg/kg/animal); G1: Grape seed oil group (1000 mg/kg/animal); G2: Grape seed oil group (2000 mg/kg/animal); GL: blend oil (2000 mg/kg/animal). Mean ± standard deviation from the mean. Different letters in the same line indicate significant differences in relation to C (*p* < 0.05: a; *p* < 0.001: A), O (*p* < 0.05: b), L1 (*p* < 0.05: c), G1 (*p* < 0.05: d), and GL (*p* < 0.05: e). Kruskal–Wallis/Dunn’s tests ^1^; ANOVA/Tukey’s tests).

**Table 8:** Distribution of changes observed in the liver of experimental animals.

Parameters	C		O		L1		L2		G1		G2		GL	
	n	%	n	%	n	%	n	%	n	%	n	%	n	%
Steatosis														
<5%	12	100	12	100	12	100	12	100	12	100	12	100	12	100
Microvesicle steatosis														
Absent	12	100	12	100	12	100	12	100	12	100	12	100	12	100
Lobular inflammation (*p* = 0.528)														
Absent/<2 foci per field/200X	12	100	12	100	11	91.7	12	100	12	100	11	91.7	12	100
2–4 foci per field/200X	0	0	0	0	1	8.3	0	0	0	0	1	8.3	0	0
Ballooning (*p* = 0.008)														
Absent/few cells	12	100	11	91.7	12	100	10	83.3	8	66.7	7	58.3 ^a,b,c^	12	100
Many cells	0	0	1	8.3	0	0	2	16.7	4	33.3	5	41.7	0	0
Mallory’s hyaline (*p* = 0.421)														
Absent	9	75	10	83.3	9	75	10	83.3	6	50	7	58.3	7	58.3
Present	3	25	2	16.7	3	25	2	16.7	6	50	5	41.7	5	41.7
Apoptosis (*p* = 0.143)														
Absent/few cells	12	100	10	83.3	12	100	8	66.7	8	66.7	9	75	10	83.3
Present	0	0	2	16.7	0	0	4	33.3	4	33.3	3	25	2	16.7
Glycogenated nuclei (*p* = 0.249)														
None/rare	8	66.7	9	75	9	75	9	75	7	58.3	11	91.7	8	66.7
Some	4	33.3	3	25	3	25	3	25	5	41.7	1	8.3	4	33.3

C: Control group (distilled water at dose of 1000 mg/kg/animal); O: extra virgin olive oil group (1000 mg/kg/animal); L1: Golden linseed oil group (1000 mg/kg/animal); L2: Golden linseed oil group (2000 mg/kg/animal); G1: Grape seed oil group (1000 mg/kg/animal); G2: Grape seed oil group (2000 mg/kg/animal); GL: Blend oil (2000 mg/kg/animal). The data are presented as absolute and relative frequencies. Different letters in the same line indicate significant differences calculated using Bonferroni correction with respect to C (*p* < 0.05: a), L1 (*p* < 0.05: b), and GL (*p* < 0.05: c).

## Data Availability

The original contributions presented in the study are included in the article, further inquiries can be directed to the corresponding author.
